# Adaptor Scaffoldins: An Original Strategy for Extended Designer Cellulosomes, Inspired from Nature

**DOI:** 10.1128/mBio.00083-16

**Published:** 2016-04-05

**Authors:** Johanna Stern, Sarah Moraïs, Raphael Lamed, Edward A. Bayer

**Affiliations:** a Department of Biological Chemistry, The Weizmann Institute of Science, Rehovot, Israel; b Department of Molecular Microbiology and Biotechnology, Tel Aviv University, Ramat Aviv, Israel

## Abstract

Designer cellulosomes consist of chimeric cohesin-bearing scaffoldins for the controlled incorporation of recombinant dockerin-containing enzymes. The largest designer cellulosome reported to date is a chimeric scaffoldin that contains 6 cohesins. This scaffoldin represented a technical limit of sorts, since adding another cohesin proved problematic, owing to resultant low expression levels, instability (cleavage) of the scaffoldin polypeptide, and limited numbers of available cohesin-dockerin specificities—the hallmark of designer cellulosomes. Nevertheless, increasing the number of enzymes integrated into designer cellulosomes is critical, in order to further enhance degradation of plant cell wall material. Adaptor scaffoldins comprise an intermediate type of scaffoldin that can both incorporate various enzymes and attach to an additional scaffoldin. Using this strategy, we constructed an efficient form of adaptor scaffoldin that possesses three type I cohesins for enzyme integration, a single type II dockerin for interaction with an additional scaffoldin, and a carbohydrate-binding module for targeting to the cellulosic substrate. In parallel, we designed a hexavalent scaffoldin capable of connecting to the adaptor scaffoldin by the incorporation of an appropriate type II cohesin. The resultant extended designer cellulosome comprised 8 recombinant enzymes—4 xylanases and 4 cellulases—thereby representing a potent enzymatic cocktail for solubilization of natural lignocellulosic substrates. The contribution of the adaptor scaffoldin clearly demonstrated that proximity between the two scaffoldins and their composite set of enzymes is crucial for optimized degradation. After 72 h of incubation, the performance of the extended designer cellulosome was determined to be approximately 70% compared to that of native cellulosomes.

## IMPORTANCE

Plant cell wall residues represent a major source of renewable biomass for the production of biofuels such as ethanol via breakdown to soluble sugars. The natural microbial degradation process, however, is inefficient for achieving cost-effective processes in the conversion of plant-derived biomass to biofuels, either from dedicated crops or human-generated cellulosic wastes. The accumulation of the latter is considered a major environmental pollutant. The development of designer cellulosome nanodevices for enhanced plant cell wall degradation thus has major impacts in the fields of environmental pollution, bioenergy production, and biotechnology in general. The findings reported in this article comprise a true breakthrough in our capacity to produce extended designer cellulosomes via synthetic biology means, thus enabling the assembly of higher-order complexes that can supersede the number of enzymes included in a single multienzyme complex.

## INTRODUCTION

Cellulosomes are discrete multienzymatic complexes secreted by anaerobic cellulolytic bacteria (1, 2). This kind of molecular machine is considered to be among the most efficient of all enzymatic systems for degradation of plant matter, due to the organization of its enzymes in close proximity that facilitates stronger synergism among the catalytic units. The cellulosome was first discovered in *Clostridium thermocellum* as a multisubunit entity of complicated quaternary structure with a size of about 18 nm (2). It presents an elementary structure based on a primary scaffoldin subunit that attaches to the substrate via a carbohydrate-binding module (CBM) and incorporates different enzymes via specific high-affinity type I cohesin-dockerin interactions. The cellulosome is attached to the bacterial cell surface via a second type (type II) of cohesin-dockerin interaction between the primary scaffoldin and an anchoring scaffoldin, which connects to the cell via an S layer homology (SLH) module (3). The resultant high-molecular-weight machine is particularly effective in degrading plant cell wall material, since it results in enzyme proximity and targeting effects which allow minimal diffusion loss of enzymes and hydrolytic products (2, 4, 5).

More elaborate cellulosomal systems were discovered later, such as the cellulosomes of *Ruminococcus flavefaciens* (6, 7), *Acetivibrio cellulolyticus* (8, 9), and *Clostridium clariflavum* (10, 11), which employ intricate networks of numerous interacting scaffoldins that amplify the number of enzymes integrated in the system. This strategy also allows more flexibility for enzyme integration and, thus, better adaptation for bacterial survival in a variety of ecosystems. These large cellulosomes can have a molecular mass of more than 100 MDa and appear clearly as protuberances on the surface of cells (12).

Designer cellulosomes are artificial nanodevices that allow controlled incorporation of plant cell wall-degrading enzymes and, thus, represent a potential platform for processing biomass into biofuels. The fabrication of designer cellulosomes was first proposed in 1994 and is based on the very high affinities (1, 13) and specific interactions (14, 15) between matching cohesin and dockerin modules, thus enabling self-assembly of their components. For this purpose, a chimeric scaffoldin is produced that, unlike native cellulosomal systems, contains cohesins of divergent specificities together with a collection of enzymes that contain matching types of dockerins. Previous studies using designer scaffoldins have resulted in enhanced activities in the hydrolysis of various recalcitrant substrates (16–18). In most of these, the configurations of the designer cellulosomes mimicked the overall simplistic architecture of the *C. thermocellum* cellulosome. Only a few studies have attempted to design more complex structural composites (19, 20).

The most elaborate designer cellulosome reported to date was designed by our group and is composed of a hexavalent chimeric scaffoldin containing 6 cohesins and a targeting CBM (21). This scaffoldin enabled the precise integration of 4 recombinant xylanases and 2 recombinant cellulases originating from the free aerobic noncellulosomal system of *Thermobifida fusca*. The hexavalent designer cellulosome provided both targeting and proximity effects, leading to enhanced wheat straw degradation compared with the efficiency of the wild-type enzyme mixture. The hexavalent cellulosome reached about 40% of the degradation efficiency of the naturally secreted cellulosome of *C. thermocellum*, considered today the most efficient system for plant cell wall degradation. Furthermore, the degradation products detected were mainly derived from the cellulosic portion of the substrate, thus indicating that more cellulases should be integrated in the cocktail. However, the length of the chimeric scaffoldin appeared to impose a technical limit, since the addition of another cohesin proved problematic. Scaffoldins containing more than six cohesins were difficult to express, and the products were fragile and unstable. Furthermore, the list of available well-characterized cohesin-dockerin specificities is currently restricted. Thus, our capacity to construct designer cellulosomes with expanded numbers of enzyme components was likewise limited.

Due to the intricate composition of the plant cell wall, its efficient degradation requires the involvement of a large number of enzymes exhibiting multiple functionalities (22). Different physical parameters, such as pH, temperature, and adsorption, and chemical factors, such as nitrogen, phosphorus, and the presence of phenolic compounds, can critically inhibit polysaccharide-degrading catalytic subunits and, thus, reduce the bioconversion of lignocellulose (23, 24). A variety of pretreatment procedures that have been developed toward cellulose biodegradation were also shown to be responsible for the release of important amounts of cellulase inhibitors (25). Hydrolysis of lignocellulose while avoiding pretreatment would thus represent an interesting approach to limit the release of such inhibitors.

In this study, we propose an original concept to further extend the scope and composition of designer cellulosomes for degradation of a crude natural substrate, wheat straw. The approach entails the application of an adaptor scaffoldin, i.e., an intermediate type of scaffoldin that can not only incorporate various enzymes but also attach to an additional scaffoldin, thereby increasing the number and types of enzymes that can be contained in the complex. This intricate architectural model was inspired by naturally occurring adaptor scaffoldins in various cellulosome-producing bacterial species (6–11). In this context, the interscaffoldin interaction involves a type II cohesin-dockerin interaction, in contrast to the type I interactions between the scaffoldins and enzymes. The use of this category of interacting scaffoldin *in vitro* has yet to be reported in the literature. By using this strategy, together with an extensive set of precise controls, we were able to distinguish between proximity and targeting effects and amplify the number of enzymes integrated into designer cellulosomes.

Based on our previous study on optimization of cellulase activity (26), we created two different forms of adaptor scaffoldins that can incorporate three recombinant *T. fusca* enzymes (the processive endoglucanase *a*-9A, exoglucanase *b*-48A, and endoglucanase 5A-*t*) and also bind to an additional modified form of the hexavalent scaffoldin that enables the integration of five additional *T. fusca* recombinant enzymes and four xylanases (43A-*c*, 11A-*a*, 10B-*t*, and 10A-*f*), as well as endoglucanase 6A-*g*. As a consequence, this configuration of an extended designer cellulosome resulted in a significant enhancement in the level of degradation of a native untreated lignocellulosic substrate.

## RESULTS

### Chimeric scaffoldin library.

**FIG 1:**
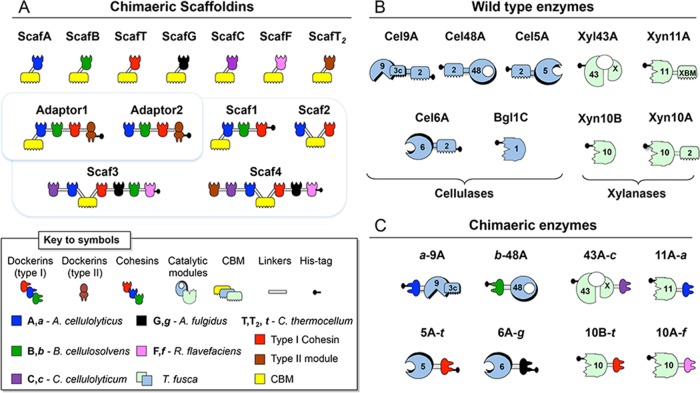
Schematic representation of the recombinant proteins. The bacterial origins of the representative modules are shown by color coding in the pictograms. In the shorthand notation for the recombinant enzymes, the numbers 9, 48, 5, 6, 1, 43, 11, and 10 correspond to the GH (glycoside hydrolase) family of the catalytic modules, uppercase characters indicate the bacterial origin of the cohesin modules, and T_2_ specifies a type II cohesin. The bacterial origin of a given dockerin module is indicated by a lowercase italic character. The wild-type *T. fusca* cellulases (except Bgl1C) and xylanase Xyn10A contain a cellulose-specific family 2 CBM. The CBMs of the chimeric scaffoldins are derived from the family 3a CBM from *C. thermocellum* CipA. Wild-type and recombinant forms of Cel9A contain a family 3c CBM.

The 13 chimeric scaffoldins employed in this study are represented schematically in Fig. 1A. We produced 7 monovalent scaffoldins, each composed of a single cohesin with different specificities and a CBM3a module from *C. thermocellum*. ScafA, ScafB, ScafT, ScafG, ScafC, and ScafF possess type I cohesin modules derived from the following microbes, as described previously (27): cohesin A from *A. cellulolyticus*, cohesin B from *Bacteroides cellulosolvens*, cohesin C from *C. cellulolyticum*, cohesin T from *C. thermocellum*, cohesin G from *Archaeoglobus fulgidus*, cohesin C from *Clostridium cellulolyticum*, and cohesin F from *R. flavefaciens*. In contrast, ScafT_2_ bears a type II cohesin module that mediates scaffoldin-to-scaffoldin interactions in the native *C. thermocellum* cellulosome.

Two adaptor scaffoldin variants were created, each possessing the same three type I cohesin modules (A, B, and T as described above) for enzyme integration and a type II dockerin (T_2_) for interaction with an additional scaffoldin that contains the matching type II cohesin. Adaptor1 possesses a CBM3a module, whereas Adaptor2 lacks a cellulose-targeting CBM.

We also produced two hexavalent scaffoldins: Scaf3, as described in a previous publication, integrates 6 dockerin-bearing enzymes, and Scaf4 is able to integrate 5 dockerin-bearing enzymes but contains a type II cohesin module (from *C. thermocellum*) that allows interaction with the type II cohesin of one of the adaptor scaffoldins described above. Both hexavalent scaffoldins possess a CBM3a module for targeting to the cellulosic substrate.

In addition, a trivalent and a divalent scaffoldin, Scaf1 and Scaf2, respectively, were employed in order to further characterize proximity and targeting effects. Both scaffoldins have been described in previous publications (16, 28). Additional information about the nomenclature and modular composition of the recombinant proteins employed here can be found in Table S1 in the supplemental material, and molecular mass data can be found in Table S2.

### Wild-type and recombinant *T. fusca* enzymes.

Schematic representations of the modular structures of the wild-type and recombinant enzymes from *Thermobifida fusca* employed in this study appear in Fig. 1B and C, respectively. Recombinant cellulosomal forms of *T. fusca* enzymes exoglucanase *b*-48A, endoglucanase 6A-*g*, and xylanases 11A-XBM-*a* and 10A-*f* were obtained as previously reported by replacing their CBM2 modules with dockerins with different specificities (21). The recombinant forms of the processive endoglucanase *a*-9A and endoglucanase 5A-*t* were produced in the inverted forms, where the CBM2 module was removed and the dockerin modules were placed on the opposite side of the protein as detailed in previous publications (16, 26). β-Xylosidase 43A-*c* and xylanase 10B-*t* were obtained by adding the relevant dockerin module at the C terminus of the protein (21). The wild-type *T. fusca* enzymes used in this study were fully characterized previously (29–32).

### Cohesin-dockerin specificity.

**FIG 2:**
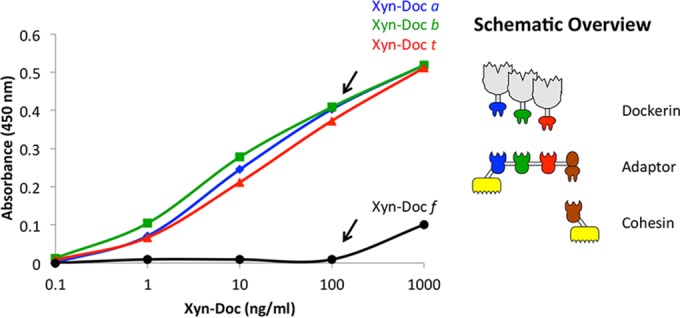
Results of modified ELISA-based system for simultaneous binding of the adaptor scaffoldin. Microtiter plates were coated with the monovalent scaffoldin ScafT_2_. The plates were then subjected to interaction with excess adaptor scaffoldin. Specified xylanase-fused dockerins were then introduced at specified amounts. Only the relevant xylanase-fused dockerins Xyn-Doc *a*, Xyn-Doc *b*, and Xyn-Doc *t* revealed appropriate binding signals, whereas the nonspecific Xyn-Doc *f* exhibited only poor interaction at a very high concentration. No signal was observed in the absence of adaptor scaffoldin (data not shown).

The specificities of both dockerin- and cohesin-containing proteins were examined semiquantitatively with affinity-based enzyme-linked immunosorbent assays (ELISAs) (27). Each module was able to bind selectively with its matching partner and showed no or very poor binding to nonmatching ones. In addition, in order to ensure that the adaptor scaffoldins were able to bind simultaneously to the recombinant enzymes via their three cohesin modules and to the chimeric scaffoldin via their dockerin type II modules, a modified ELISA-based assay was performed (Fig. 2). Microtiter plates were first coated with the monovalent scaffoldin ScafT_2_ and then by the desired adaptor scaffoldin. Finally, the three different xylanase-fused dockerins were introduced in specified amounts. The results indicated species-specific selective binding of the dockerin-bearing xylanase to the respective cohesin partner, relative to each other and to an independent control.

### Analysis of complex formation.

**FIG 3:**
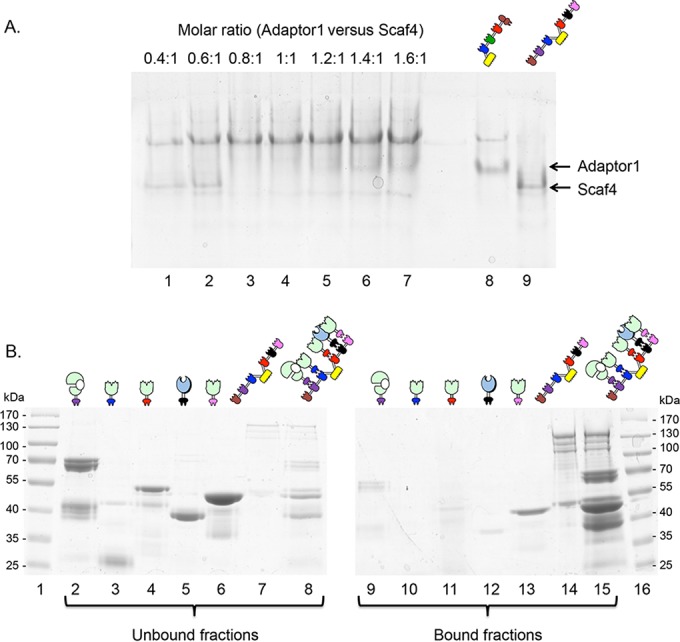
Analysis of complex formation. (A) Results of nondenaturing PAGE for interaction between Adaptor1 and Scaf4. Lanes 1 to 7 correspond to complexes of the two scaffoldins at different ratios. Lane 8 corresponds to Adaptor1 alone, and lane 9 corresponds to Scaf4 alone (100 pmol each). For all gels, the amount of Scaf4 was fixed at 100 pmol, and the amount of Adaptor1 was adjusted according to the molar ratio specified. (B) Results of affinity pulldown assay. Chimeric enzymes and scaffoldin were assayed individually for binding to a cellulosic substrate. Enzymes and scaffoldin were then mixed at equimolar ratios and introduced to a cellulosic substrate (Avicel). The cellulose-binding abilities of individual proteins and the resultant complex were determined by examining the unbound (lanes 2 to 8) and cellulose-bound (lanes 9 to 15) fractions by SDS-PAGE. Lanes 1 and 16, molecular mass markers. Lanes 2 to 8 contain unbound fractions as follows: lane 2, 43A-*c*; lane 3, 11A-*a*; lane 4, 10B-*t*; lane 5, 6A-*g*; lane 6, 10A-*f*; lane 7, Scaf4; lane 8, complex of the six proteins. Lanes 9 to 15 contain bound fractions of the proteins in the same order. In the presence of the chimeric scaffoldin, the enzymatic components were associated with the cellulose-bound fraction, whereas in its absence, they remained in the unbound fraction.

Stoichiometric ratios for relevant matching protein couples were determined by nondenaturing PAGE assays. When both proteins were combined at the exact stoichiometric ratio, a single major band with altered mobility was usually observed. An example showing the binding of the hexavalent scaffoldin Scaf4 with Adaptor1 is provided in Fig. 3A.

Following nondenaturing PAGE assays, affinity pulldown analysis was performed. For this purpose, stoichiometric amounts of selected dockerin-bearing enzymes were combined with a chimeric scaffoldin that contained the appropriate matching cohesins (see an example in Fig. 3B). All recombinant proteins exhibited single major bands on SDS-polyacrylamide gels, with mobility patterns consistent with their molecular mass. The resultant complexes, as well as samples containing the individual recombinant enzymes alone, were allowed to interact with microcrystalline cellulose. Samples were then centrifuged to separate bound and unbound fractions, and the fractions were subjected to SDS-PAGE. Individual enzymes appeared in the unbound fractions, whereas scaffoldin-complexed enzymes were detected in the bound fractions, thus indicating that the complex was formed at correct equimolar ratios (see Fig. S1 in the supplemental material).

### Avicel degradation using adaptor scaffoldins alone.

**FIG 4:**
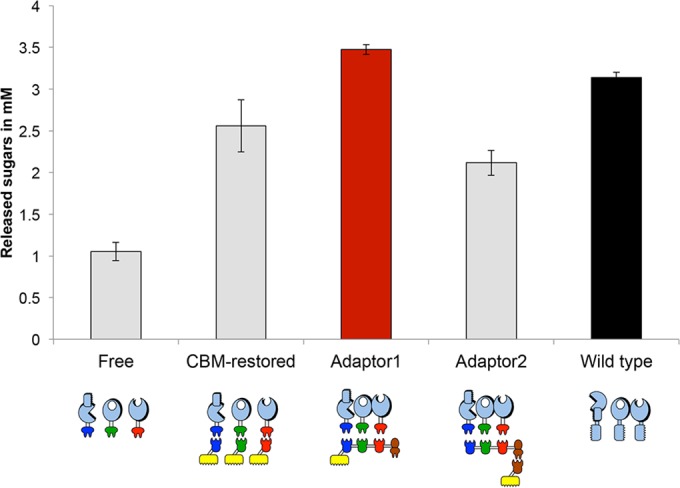
Comparative degradation of Avicel by the various complexes and free enzyme systems after 48 h. The compositions of the complexes and free enzyme systems are indicated by pictograms. The red bar shows the results for the optimal configuration, while the black bar shows the results for the wild-type enzyme mixture. Enzymatic activity is defined as total reducing sugars released (mM). Error bars show standard deviations.

We tested the cellulolytic activities of the three recombinant enzymes *a*-9A, *b*-48A, and 5A-*t* in combination with 2 different forms of adaptor scaffoldin, namely, Adaptor1, which possesses a CBM3a module, and Adaptor2, which does not (Fig. 4). The adaptor scaffoldin that lacks a CBM, Adaptor2, was also targeted to the substrate, but indirectly, via interaction with a relevant monovalent scaffoldin, ScafT_2_. As in previous studies (26, 28, 33), microcrystalline Avicel, a relatively complex model cellulosic substrate, was chosen for degradation, since it could enable distinction of the targeting and proximity effects conferred by the various designer cellulosomal compositions. We observed that this indirect type of substrate targeting (using Adaptor2 and ScafT_2_) was much less efficient than direct targeting of the enzymes using the CBM-bearing adaptor scaffoldin (Adaptor1). Only by using the CBM-containing adaptor were we able to observe both the proximity and targeting effects of the enzymatic combinations that resulted in relatively low but significant improvements compared with the performance of the wild-type mixture. This form of adaptor scaffoldin was thus selected for the construction of extended designer cellulosomes by interaction with the hexavalent scaffoldin Scaf4.

### Performance of the extended designer cellulosome in wheat straw degradation.

**FIG 5:**
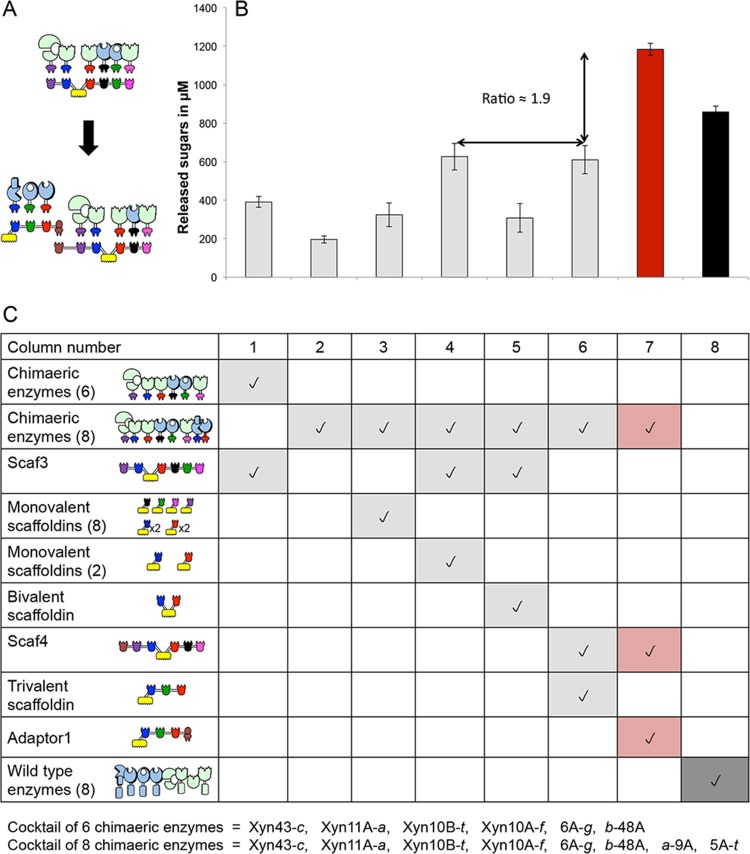
Extended designer cellulosomes. (A) Schematic representation of the extension of the designer cellulosome concept. The previously described hexavalent scaffoldin with its 6 enzymes (1) is supplemented with 2 additional enzymes using the adaptor strategy, as follows: Adaptor1 integrates 3 cellulases, *a*-9A, *b*-48A, and 5A-*t*, and attaches to Scaf4, which incorporates 5 additional enzymes, including 4 xylanases, 43A-*c*, 11A-*a*, 10B-*t*, and 10A-*f*, and one cellulase, 6A-*g*. (B) Comparative degradation of wheat straw after 48 h by the chimeric cellulosomal enzymatic complexes and free chimeric and wild-type enzymes indicated in the respective columns in panel C below. Enzymatic activities, measured in total reducing sugars released (µM) are shown. Error bars show standard deviations. (C) Contents of enzymes and scaffoldins in the various samples. The enzymatic system summarized in column 1 contains only 6 chimeric enzymes, 43A-*c*, 11A-*a*, 10B-*t*, 6A-*g*, 10A-*f*, and *b*-48A, while in the systems summarized in columns 2 to 7, two additional enzymes are included, *a*-9A and 5A-*t*, to complete a total of 8 enzymes. The system summarized in column 8 is a mixture of the 8 equivalent wild-type *T. fusca* enzymes (free native system).

The assembly of selected cellulases and xylanases into an extended designer cellulosome by virtue of the involvement of a CBM-bearing adaptor scaffoldin resulted in significantly enhanced activity on a native nonpretreated substrate, wheat straw. The results are documented in Fig. 5. We assayed the wheat straw degradation profile of our resultant extended designer cellulosome, composed of the adaptor scaffoldin Adaptor1 containing the 3 recombinant cellulases *a*-9A, *b*-48A, and 5A-*t* in complex with the hexavalent Scaf4, displaying the 4 recombinant xylanases 43A-*c*, 11A-*a*, 10B-*t*, and 10A-*f*, as well as the recombinant endoglucanase 6A-*g* (Fig. 5A). Thus, eight different enzymes (4 cellulases and 4 xylanases) were assembled into a single designer cellulosome complex. In comparison with our previous study using the hexavalent scaffoldin Scaf3 (21), in the present work, the 6-enzyme cocktail was enriched with two additional cellulases: the processive endoglucanase *a*-9A and endoglucanase 6A-*g*. We compared the enzymatic activity of our extended designer cellulosome to those of a wide variety of controls, in order to assess the contribution of each individual component (Fig. 5B).

Column 1 in Fig. 5C summarizes the components of hexavalent scaffoldin Scaf3, containing xylanases 43A-*c*, 11A-*a*, 10B-*t*, and 10A-*f* and cellulases *b*-48A and 6A-*g*; the degradation assay results are shown by the first bar of Fig. 5B above. The only difference between the enzyme composition in the present work and that of our previous publication is the substitution of the recombinant form of endoglucanase Cel6A instead of endoglucanase Cel5A, both of which are known to have similar activities (34); this was possible because 5A-*t* was included in Adaptor1, and it allowed us to integrate an additional enzyme. In the mixtures whose contents are summarized in columns 4 and 5 of Fig. 5C, the 2 recombinant cellulases *a*-9A and 5A-*t* were added externally into the mixture (i.e., not as part of the extended designer cellulosome), targeted via individual monovalent scaffoldins (column 4) or a divalent scaffoldin (column 5). An increased level of degradation (about 1.6-fold) was observed by including two monovalent scaffoldins (Fig. 5B, fourth bar, and C, column 4), whereas the divalent scaffoldin Scaf2, which put *a*-9A and 5A-*t* in close proximity, appeared to create an anti-proximity effect between the two enzymes, thus leading to reduced activity. In the cellulosome summarized in column 6 of Fig. 5C, we employed the hexavalent scaffoldin Scaf4 containing xylanases 43A-*c*, 11A-*a*, 10B-*t*, and 10A-*f* and endoglucanase 6A-*g* together with an unattached trivalent scaffoldin, Scaf1. The level of degradation (Fig. 5B, sixth bar) was similar to that obtained with the combination summarized in Fig. 5C, column 4.

Using the complete extended octavalent cellulosome system, summarized in Fig. 5C, column 7, we observed the substantial effect of Adaptor1 on the efficiency of the hexavalent scaffoldin Scaf4, resulting in 1.9-fold enhancement of degradation compared to the levels achieved by the mixtures summarized in columns 4 and 6 (Fig. 5B, fourth, sixth, and seventh bars) and about 3-fold enhancement compared to the level obtained with the hexavalent scaffoldin and its 6 enzymes (Fig. 5B, first bar, and C, column 1). Furthermore, the levels achieved by the octavalent system represent a 1.4-fold increase compared to the performance of the corresponding 8 free wild-type enzymes (Fig. 5B, eighth bar, and C, column 8). This result is of major importance, since it clearly demonstrates that the advantage of the designer cellulosome strategy could be observed only when the two chimeric scaffoldins (adaptor and hexavalent scaffoldins) were linked, thereby bringing into close proximity all the different catalytic subunits.

**FIG 6:**
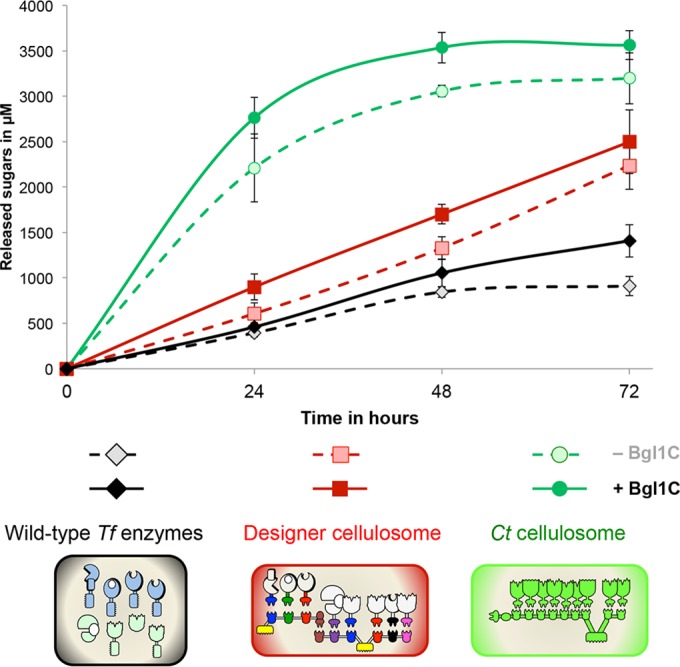
Kinetics studies of wheat straw hydrolysis by the free wild-type enzyme, extended designer cellulosome, and native cellulosome systems. The system used for degradation is identified by color and symbol as shown in the key. *Ct* cellulosome, *C. thermocellum* cellulosome; Designer cellulosome, extended designer cellulosome; Wild-type *Tf* enzymes, eight free wild-type enzymes from *T. fusca*; Bgl1C, *T. fusca* β-glucosidase. Enzymatic activity is defined as total reducing sugars released (µM). Error bars show standard deviations.

The kinetics of wheat straw degradation of the extended designer cellulosome was compared to that of the natural secreted cellulosome of *C. thermocellum* and wild-type enzymes of *T. fusca* in the presence and absence of *T. fusca* β-glucosidase Bgl1C (Fig. 6). For each enzymatic composition, Bgl1C serves to further enhance wheat straw degradation by relieving cellobiose inhibition of the enzymatic subunits. The β-glucosidase was supplemented to the reaction mixture in the free state (not bound to a scaffoldin). The following high yields of degradation (based on cellulose/hemicellulose content) were reached after a 72-h incubation at 50°C: *C. thermocellum* cellulosome, 54%; designer cellulosome, 38%; and wild-type enzymes, 21%. The performance following assembly into the extended designer cellulosome thus reached 70% of the level of wheat straw degradation by the native *C. thermocellum* cellulosome. Moreover, after 72 h, degradation by the designer cellulosome appeared to be in the linear phase, while that of the *C. thermocellum* cellulosome had reached saturation.

### Soluble sugar production.

Analysis of soluble sugar production revealed that the assembly of enzymes into the extended designer cellulosome allowed the production of larger amounts of all sugars than were produced by the wild-type enzyme cocktails (with the exception of arabinose in the absence of Bgl1C). When β-glucosidase was not present (free state), no glucose was produced either from the action of designer cellulosomes or wild-type enzymes, whereas the *C. thermocellum* cellulosome produced relatively large amounts of glucose. In this context, the broad range of cellulases in the cellulosome preparation (35–37), many of them uncharacterized, could lead to glucose production, as opposed to the limited types of *T. fusca* enzymes included in the designer cellulosome and parallel wild-type enzyme cocktail. On the other end, the enzymatic composition of the extended designer cellulosome appeared to be more efficient in terms of xylan degradation. In most samples, arabinose was also detected, presumably as a residual by-product, independent of enzyme content (Table 1).

**TABLE 1:** Soluble sugar production from digestion of hatched wheat straw during a 72-h incubation by various enzyme combinations

Enzyme combination	Amt released (µM) ± SD (mg/g of substrate)					
	Glucose	Cellobiose	Cellotriose	Xylose	Xylotriose	Arabinose
Free wild-type enzymes (8 enzymes)	ND^a^	198 ± 11 (34)	ND	428 ± 20 (32)	ND	375 ± 7 (28)
Hexavalent scaffoldin + adaptor scaffoldin	ND	264 ± 21 (45)	ND	903 ± 74 (68)	ND	237 ± 0.3 (18)
*C. thermocellum* cellulosome	1,159 ± 18 (104)	358 ± 11 (61)	ND	346 ± 24 (26)	ND	286 ± 5 (21)
Free wild-type enzymes (8 enzymes) + Bgl1C	898 ± 1 (81)	79 ± 4 (13)	ND	302 ± 23 (23)	ND	ND
Hexavalent scaffoldin + adaptor scaffoldin + bgl1c	1,021 ± 10 (92)	71 ± 3 (12)	ND	654 ± 74 (49)	ND	237 ± 2 (18)
*C. thermocellum* cellulosome + Bgl1C	1,444 ± 31 (130)	33 ± 4 (6)	ND	431 ± 8 (32)	ND	268 ± 4 (20)

a ND, not detected.

## DISCUSSION

The development of the designer cellulosome concept has advanced in a slow and incremental but consistent fashion over the past two decades. From its initial conception in 1994 (1), the first concrete experimental attempts in 2001 progressed with the successive fabrication of monovalent (38), divalent (16, 5), trivalent (28, 33), tetravalent (18), and ultimately, hexavalent (21) scaffoldins for the production of larger and more intricate forms of designer cellulosome systems. Likewise, early work that focused on cellulase-mediated degradation of pure cellulosic substrates in designer cellulosome formats (38, 5, 33) subsequently progressed to the inclusion of xylanases (18, 21, 33, 39) and oxidative enzymes and their combined action on native forms of plant-derived substrates (40).

Nevertheless, appending an additional cohesin to the scaffoldin sequence represents a major drawback in this endeavor, since extended scaffoldins become unstable, difficult to express with regular *Escherichia coli* tools, and otherwise restricted due to the limited number of well-characterized cohesin-dockerin specificities available. However, increasing the number of enzymatic subunits integrated is critical for enhancing the degradation of plant cell wall residues and rendering the production of biofuels cost effective. In this context, additional expansion of the enzymatic repertoire of designer cellulosomes to encompass other types of both enzymatic and nonenzymatic components is warranted in order to further amplify the overall hydrolytic capacity of designer cellulosomes on lignocellulosic biomass.

In this study, we explored an original strategy to create more complex designer cellulosomes by employing an adaptor scaffoldin, namely, a bifunctional chimeric scaffoldin that is able both to incorporate enzymes via several copies of type I cohesins and to attach to an additional scaffoldin via a type II dockerin. We discovered during this work that in order to achieve an efficient form, the adaptor has to possess a CBM as an inherent part of its amino acid sequence. This finding contrasts with the results of early work, which indicated that the presence of more than one cellulose-binding CBM per designer cellulosome complex interferes with cellulose-degrading activity (19, 38). Those studies, however, involved relatively small designer cellulosome complexes. One possible explanation, therefore, would be that the additional CBM facilitates interaction with the substrate and the subsequent performance of the extended complexes used in this work. In any case, our present results demonstrate that the previously assumed limitation should not necessarily be considered a rule for further design of interacting chimeric scaffoldins.

The content of the efficient adaptor scaffoldin corresponds to native forms of enzyme-bearing scaffoldins, i.e., multiple type I cohesin modules, a type II dockerin, and a CBM. Except in certain ruminococcal species, scaffoldins that mediate enzyme integration generally possess an intrinsic CBM. In nature, two types of adaptor scaffoldin have been defined. The first corresponds to an intermediate scaffoldin that integrates multiple copies of the enzyme-bearing scaffoldin, leading to substantial amplification of the number of enzymes included in a single cellulosomal complex, as exemplified by ScaB of *A. cellulolyticus* or *C. clariflavum* (9, 11). The second type provides a mechanism for changes in the repertoire of cellulosomal enzymatic subunits. Such a scaffoldin is generally monovalent and enables the incorporation of a class of dockerin-containing proteins into the cellulosome complex which are not recognized directly by the major enzyme-bearing scaffoldin (7, 41). In this work, we were inspired by these two natural models of adaptor scaffoldin to design a chimeric adaptor scaffoldin which enables amplification of both the number and diversity of catalytic subunits incorporated into the system.

In the current study, the adaptor scaffoldin served to integrate three recombinant cellulases from *T. fusca*, i.e., processive endoglucanase *a*-9A, exoglucanase *b*-48A, and endoglucanase 5A-*t*, in order to extend the number of enzymes that could be integrated into a designer cellulosome assembly. Based on our previously published findings on the importance of the location of enzymes in chimeric scaffoldins (26), we employed the same concept, which indicated that the *a*-9A processive endoglucanase should be in close proximity to the CBM module. We thus propose that the presence of the CBM module in the adaptor scaffoldin is a determining factor that may contribute to enhanced activity. In early attempts at designing an adaptor scaffoldin (42), we utilized a different enzymatic cocktail composed of three *T. fusca* enzymes originating from endoglucanases Cel6A and Cel5A and exoglucanase Cel48A. In that study, two different forms of adaptor scaffoldin were created, both lacking a CBM module in their amino acid sequences. Neither provided both proximity and targeting effects.

The numerous controls employed in this work allowed us to differentiate between the proximity and targeting effects of the extended designer cellulosome. We thus observed that the final extended octavalent designer cellulosome exhibited enhanced degradation capacity compared to the activity of a mixture of smaller designer cellulosomes with the same enzyme content. We also observed the relevance of adding two recombinant cellulases to the enzymatic cocktail: the processive endoglucanase *a*-9A, a key enzyme of the *T. fusca* system, and endoglucanase 6A-*g*. This supports previous observations that, for optimal degradation of crude plant-derived materials, a higher ratio of cellulases versus xylanases should be used (43). Indeed, cellulases represent a major part of natural cellulosomes relative to the proportions of other glycoside hydrolases (35–37). We observe here that this addition could not be performed randomly: when *a*-9A and 5A-*t* were integrated into a divalent scaffoldin and included with the hexavalent scaffoldin, no gain in activity was observed over that of the hexavalent scaffoldin alone. This further supports our previous finding on enzyme location that these two enzymes should not be placed next to each other, presumably due to a functional anti-proximity effect (26). Careful addition of other cellulases may be considered for an optimal ratio between xylanases and cellulases.

Another interesting strategy (20) employed increased complexity of designer cellulosomes by producing recombinant dockerin-bearing multifunctional enzymes that could be integrated into chimeric scaffoldins. High levels of cellulose degradation were obtained, taking advantage of intramolecular synergy between the multiple catalytic modules present in single gene products. Multifunctional enzymes are present in various anaerobic microorganisms, both in cellulosome-producing bacteria (10, 35, 44, 45) and in free-enzyme-producing hyperthermophilic bacteria (46, 47). A combination of both strategies (i.e., adaptor and multifunctional enzymes) could represent a valuable future development for designer cellulosomes.

The aerobic *T. fusca* polysaccharide-degrading system offers additional possibilities that are not achievable by natural cellulosomal anaerobic systems, i.e., oxidative tools which play a role in rendering lignocellulosic substrates more accessible to (hemi)cellulase-mediated hydrolysis (48, 49). A previous study from our laboratory demonstrated a dramatic boosting effect of adding lytic polysaccharide monooxygenases (LPMOs) into designer cellulosomes (40) and suggested possible advantages of adding laccases (to remove inhibition by phenols) into the system (50, 51). Hence, using recombinant *T. fusca* enzymes in designer cellulosomes represents a powerful approach, since it allows us to combine the advantages of both anaerobic (cellulosome) and aerobic systems.

The adaptor strategy presented here opens the door for the synthesis and production of exciting forms of complex designer cellulosome structures that could comprise critical components of very efficient enzymatic cocktails for enhanced degradation of natural lignocellulosic substrates *en route* to cost-effective production of biofuels.

## MATERIALS AND METHODS

### Cloning.

Cloning of the wild-type enzymes (Cel48A, Cel5A, Cel6A, Xyl43A, Xyn11A, Xyn10B, and Xyn10A), the chimeras (*b*-48A, 5A-*t*, 43A-*c*, 11A-XBM-*a*, 10B-*t* and 10A-*f*), and the recombinant scaffoldins (ScafA, ScafB, ScafT, ScafG, ScafC, ScafF, ScafT_2_, Scaf1, Scaf2, and Scaf3) was performed as described previously (18, 21, 28, 31, 39, 52, 53). The wild-type Cel9A enzyme was a generous gift of David Wilson (Cornell University, Ithaca, NY), and the chimeric form *a*-9A was synthesized as described recently (26).

Recombinant DNA for the desired chimeric proteins was obtained by standard restriction-based cloning procedures (54). A plasmid for the chimeric enzyme 6A-*g* was created by fusing the catalytic module of the *T. fusca* endoglucanase Cel6A to a dockerin from *A. fulgidus* (Orf2375). Plasmids for adaptor scaffoldins with or without a CBM3a, i.e., Adaptor1 and Adaptor2 (see Fig. 1 for terminology), were obtained by integrating fragments including CBM3a and cohesins A, B, and T for Adaptor1 and cohesins A, B, and T alone for Adaptor2 next to the type II dockerin from the *C. thermocellum* CipA scaffoldin. The hexavalent scaffoldin Scaf4 was obtained by fusing a *C. thermocellum* type II cohesin (from anchoring scaffoldin OlpB) to a fragment that included cohesins C and A, CBM3a, and cohesins T, G, and F (primers are listed in Table S3 in the supplemental material).

PCRs were performed using Phusion high-fidelity DNA polymerase F530-S (New England Biolabs, Inc.), and DNA samples were purified using a HiYield gel/PCR fragment extraction kit (Real Biotech Corporation, RBC, Taipei, Taiwan).

### Protein expression and purification.

The production of most of the recombinant proteins used in this study has been described previously (18–20, 22). The plasmids encoding 6A-*g* and Adaptor2 were expressed in *Escherichia coli* BL21(λDE3)pLysS cells, and the proteins were purified on a Ni-nitrilotriacetic acid (NTA) column (Qiagen) as reported previously (55). The plasmids encoding Adaptor1 and Scaf4 were expressed, and the proteins were purified on phosphoric acid-swollen cellulose (PASC) (7.5 mg ml^−1^, pH 7), according to a previously described methodology (28, 56). The purity of the recombinant proteins was tested by SDS-PAGE on 12% acrylamide gels. Protein concentration was estimated from the absorbance at 280 nm based on the known amino acid composition of the protein using the Protparam tool (http://www.expasy.org/tools/protparam.html). Proteins were stored in 50% (vol/vol) glycerol at −20°C.

### Affinity-based ELISA.

The matching fusion protein procedure of Barak et al. (27) was followed to determine cohesin-dockerin specificities.

### Nondenaturing PAGE.

The extent of interaction and exact equimolar ratios between the cohesin-bearing scaffoldin and dockerin-bearing enzymes were determined by differential mobility using nondenaturing PAGE. Protein samples (4 to 8 µg each) were added to Tris-buffered saline (TBS) (pH 7.4) supplemented with 10 mM CaCl_2_ and 0.05% Tween 20 to a total volume of 30 µl. The tubes were incubated for 2 h at 37°C. Sample buffer (7.5 µl in the absence of SDS) was added to 15-µl amounts of the reaction mixtures, and the samples were loaded onto nondenaturing gels (4.3% stacking gels–9% separating gels).

### Affinity pulldown assays.

Equimolar amounts of pure proteins were prepared (100 pmol each in 50 mM acetate buffer [pH 5.0], 12 mM CaCl_2_, 2 mM EDTA) and incubated for 2 h at 37°C in the presence of 10% cellobiose (Sigma-Aldrich Chemical Co., St. Louis, MO). Cellobiose binds to the catalytic modules of the enzymes and then blocks the binding and action of the enzymes on the cellulosic substrate. The fractions were then gently mixed with microcrystalline cellulose (Avicel; FMC Biopolymer, Philadelphia, PA) for 1 h at 4°C. The tubes were centrifuged at 16,000 × *g* for 2 min. The supernatant fluids (containing unbound proteins) were carefully removed and supplemented with SDS-containing buffer to a final volume of 60 µl. The pellets (containing bound proteins) were washed twice by resuspension in 200 µl of 50 mM acetate buffer supplemented with 0.05% Tween 20 to eliminate nonspecific binding. The samples were then centrifuged at 16,000 × *g* for 2 min and resuspended in 60 µl of SDS-containing buffer (New England BioLabs). The resultant unbound and bound fractions were each boiled for 10 min and then analyzed by SDS-PAGE using a 10% polyacrylamide gel.

### Assembly of designer cellulosomes.

The various components of the designer cellulosomes (enzymes and scaffoldins) were mixed at predetermined equimolar ratios (concentration of 0.3 µM for each component) and were allowed to interact for 2 h in the presence of 10 mM CaCl_2_. For the assembly of the designer cellulosomes containing an adaptor scaffoldin, each scaffoldin was allowed to interact with its relevant enzymes separately for 2 h. Once complexes were formed, the two scaffoldins (laden with enzymes) were mixed together and subjected to interaction for an additional 2 h.

### Enzymatic activity.

The crystalline cellulosic substrate Avicel (FMC Biopolymer, Philadelphia, PA), in 80-µl amounts of a 10% solution (wt/vol), was degraded by different enzymatic combinations (concentration of 0.4 µM in the predetermined optimized equimolar ratio) in a final volume of 300 µl (50 mM acetate buffer [pH 5.0], 12 mM CaCl_2_, 2 mM EDTA). Suspensions were incubated at 50°C for 48 h.

Hatched wheat straw (Valagro, Poitiers, France) was not pretreated but only blended to a final range of lengths of about 0.2 to 0.8 mm as described previously (39). A typical assay mixture consisted of the enzymes at about 0.3 µM in a final reaction mixture volume of 400 µl (50 mM citrate buffer [pH 6.0], 12 mM CaCl_2_, 2 mM EDTA, 2 g/liter wheat straw). Where specified above, Bgl1C enzyme was added separately at 0.3 µM. Reaction mixtures were incubated at 50°C for 24 to 72 h.

*C. thermocellum* cellulosome preparations were kindly provided by Designer Energy (Rehovot, Israel) and assayed on wheat straw as described above. The final amount of cellulosomes equivalent to 0.3 µM of the extended designer cellulosome (hexavalent plus adaptor scaffoldin plus 8 enzymes) was calculated to be 241.5 µg.

Each reaction was performed in triplicate. Samples were shaken for the duration of the incubation. Reactions were terminated by immersing the sample tubes in ice water, after which the samples were centrifuged at maximum speed to remove the substrate. The total amount of sugars released was determined using the dinitrosalicylic acid (DNS) assay as described previously (57).

### Sugar analysis.

The sugars produced by the extended designer cellulosome, the wild-type enzyme mixture, and the secreted *C. thermocellum* cellulosome were analyzed by high-performance liquid chromatography (HPLC) (Agilent Technology 1260 Infinity fitted with a refractive index detector; Agilent Technology, CA) after 72 h of incubation in the presence or absence of Bgl1C. The column (300- by 7.8-mm Aminex HPX-87H; Bio-Rad, Hercules, CA) was eluted with 5 mM sulfuric acid at a flow rate of 0.6 ml/min at 45°C. Calibration curves (0 to 20 mM of glucose, cellobiose, cellotriose, xylose, xylobiose, xylotriose, or arabinose) served to determine the amounts of sugars.

## SUPPLEMENTAL MATERIAL

Figure S1  Affinity pulldown assay. All chimeric enzymes and scaffoldins were first assayed individually for binding to a cellulosic substrate. Relevant enzymes and scaffoldins were then mixed together at equimolar ratios and subsequently introduced to a cellulosic substrate (Avicel). The cellulose-binding abilities of both individual proteins and the resultant complexes were determined by examining the cellulose-unbound (lanes 2 to 6) and cellulose-bound (lanes 7 to 11) fractions by SDS-PAGE. Lanes 1 and 12, molecular mass markers. Lanes 2 to 6, unbound fractions with the following details: lane 2, *a*-9A; lane 3, *b*-48A; lane 4, 5A-*t*; lane 5, Adaptor1; lane 6, complex of *a*-9A, *b*-48A, 5A-*t*, and Adaptor1. Lanes 7 to 11, bound fractions as follows: lane 7, *a*-9A; lane 8, *b*-48A; lane 9, 5A-*t*; lane 10, Adaptor1; lane 11, complex of *a*-9A, *b*-48A, 5A-*t*, and Adaptor1. In the presence of the chimeric scaffoldin, the enzymatic components were associated with the cellulose-bound fraction, whereas in its absence, they remained in the unbound fraction. Download Figure S1, DOCX file, 0.3 MB

Table S1  Names, origins, and descriptions of modules. Table S1, DOCX file, 0.02 MB

Table S2  Molecular masses of the recombinant proteins used in this study. Table S2, DOCX file, 0.02 MB

Table S3  Primer appendix. Table S3, DOCX file, 0.02 MB
