# Supplementary material for: Adaptor Scaffoldins: An Original Strategy for Extended Designer Cellulosomes, Inspired from Nature
Source: mBio. 2016 Apr 5;7(2):e00083-16. doi: 10.1128/mBio.00083-16 (PMC4959524; doi:10.1128/mBio.00083-16)
Supplement: Figure S1 — Affinity pulldown assay. All chimeric enzymes and scaffoldins were first assayed individually for binding to a cellulosic substrate. Relevant enzymes and scaffoldins were then mixed together at equimolar ratios and subsequently introduced to a cellulosic substrate (Avicel). The cellulose-binding abilities of both individual proteins and the resultant complexes were determined by examining the cellulose-unbound (lanes 2 to 6) and cellulose-bound (lanes 7 to 11) fractions by SDS-PAGE. Lanes 1 and 12, molecular mass markers. Lanes 2 to 6, unbound fractions with the following details: lane 2, a-9A; lane 3, b-48A; lane 4, 5A-t; lane 5, Adaptor1; lane 6, complex of a-9A, b-48A, 5A-t, and Adaptor1. Lanes 7 to 11, bound fractions as follows: lane 7, a-9A; lane 8, b-48A; lane 9, 5A-t; lane 10, Adaptor1; lane 11, complex of a-9A, b-48A, 5A-t, and Adaptor1. In the presence of the chimeric scaffoldin, the enzymatic components were associated with the cellulose-bound fraction, whereas in its absence, they remained in the unbound fraction. Download [file mbo002162726sf1.docx]

**
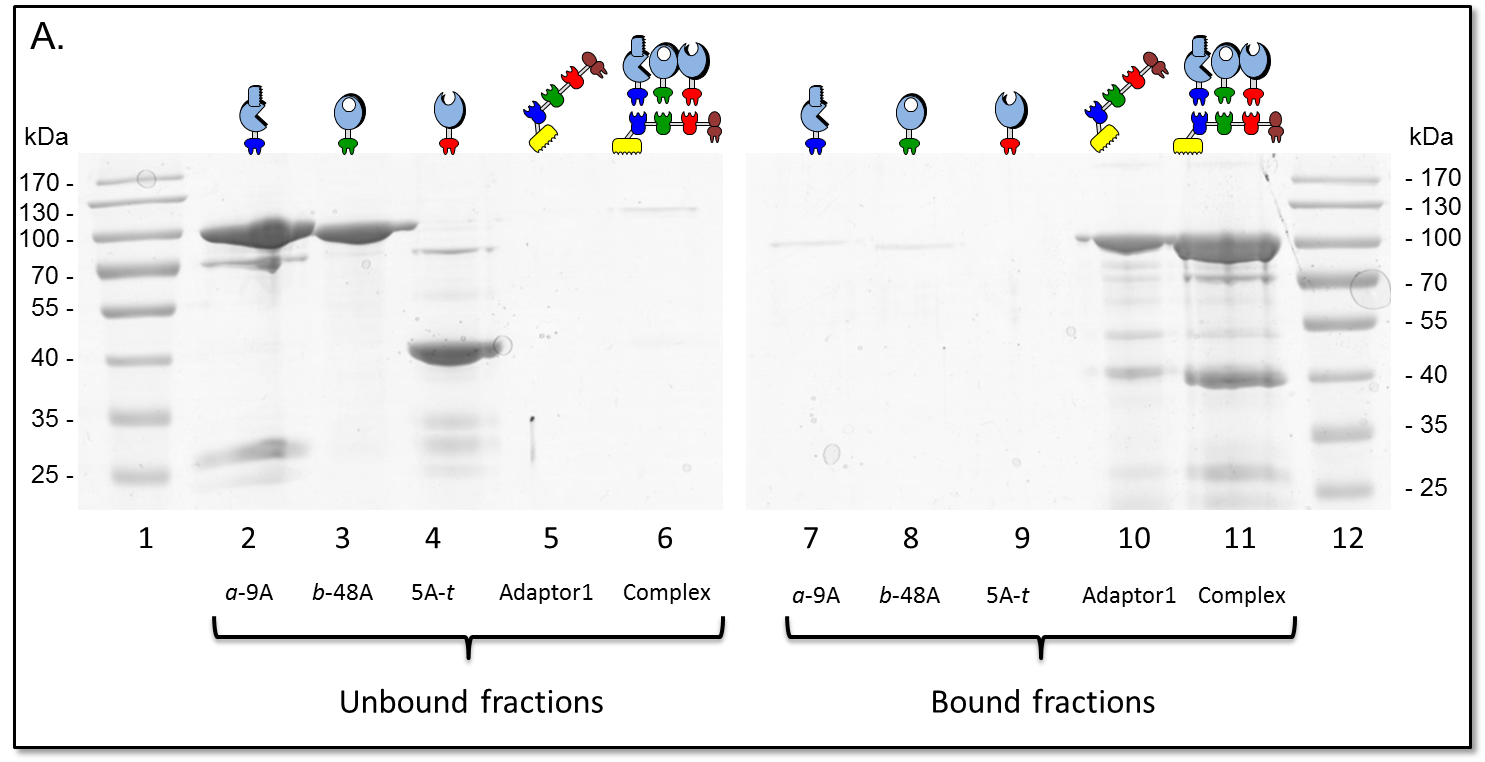
**

**Fig. S1. Affinity pull-down assay.**

All chimaeric enzymes and scaffoldins were first assayed individually for binding to a cellulosic substrate. Relevant enzymes and scaffoldin were then mixed together at equimolar ratios and subsequently introduced to a cellulosic substrate (Avicel). The cellulose-binding ability of both individual proteins and the resultant complex was determined by examining the cellulose-unbound (lanes 2-6) and bound (lanes 7-11) fractions by SDS-PAGE. Samples include: lane 1 and 12, molecular weight markers. Lane 2 to lane 6, unbound fractions with the following details: lane 2, *a*-9A; lane 3, *b*-48A; lane 4, 5A-*t*; lane 5, Adaptor1; lane 6, complex of *a*-9A, *b*-48A, 5A-*t* and Adaptor1. Lane 7 to lane 11 are bound fractions: lane 7, *a*-9A; lane 8, *b*-48A; lane 9, 5A-*t*; lane 10, Adaptor1; lane 11, complex of *a*-9A, *b*-48A, 5A-*t* and Adaptor1. In the presence of the chimaeric scaffoldin, the enzymatic components were associated with the cellulose-bound fraction; whereas in its absence, they remained in the unbound fraction.
