# Supplementary material for: Adaptor Scaffoldins: An Original Strategy for Extended Designer Cellulosomes, Inspired from Nature
Source: mBio. 2016 Apr 5;7(2):e00083-16. doi: 10.1128/mBio.00083-16 (PMC4959524; doi:10.1128/mBio.00083-16)
Supplement: Table S1 — Names, origins, and descriptions of modules. [file mbo002162726st1.docx]

**Table S1**: Modules – Name, origin and description

| **Abbreviation** ^a^ | **Module** ^b^ | **Protein source** | | **Species source** |
| --- | --- | --- | --- | --- |
|  |  | **Name** | **Description** |  |
| **A** | Cohesin C3 | ScaC | Anchoring scaffoldin | *Acetivibrio cellulolyticus* |
| **B** | Cohesin B3 | ScaB | Anchoring scaffoldin | *Bacteroides cellulosolvens* |
| **C** | Cohesin C1 | CipC | Primary scaffoldin | *Clostridium cellulolyticum* |
| **F** | Cohesin B1 | ScaB | Scaffoldin | *Ruminococcus flavefaciens* |
| **G** | Cohesin 75 | Orf2375 | Putative protein | *Archeaoglobus fulgidus* |
| **T** | Cohesin A3 | CipA | Primary scaffoldin | *Clostridium thermocellum* |
| **T_2_** | Cohesin O1 | OlpB | Anchoring scaffoldin | *Clostridium thermocellum* |
|  |  |  |  |  |
| ***a*** | Dockerin B | ScaB | Primary scaffoldin | *Acetivibrio cellulolyticus* |
| ***b*** | Dockerin A | CipBc (ScaA) | Primary scaffoldin | *Bacteroides cellulosolvens* |
| ***c*** | Dockerin A | Cel5A | Endoglucanase | *Clostridium cellulolyticum* |
| ***f*** | Dockerin A | ScaA | Scaffoldin | *Ruminococcus flavefaciens* |
| ***g*** | Dockerin 75 | Orf2375p | Putative protein | *Archeaoglobus fulgidus* |
| ***t*** | Dockerin S | Cel48S | Exoglucanase | *Clostridium thermocellum* |
| ***t_2_*** | X-Dockerin | CipA | Primary scaffoldin | *Clostridium thermocellum* |
|  |  |  |  |  |
| **1C** | GH1 | Cel5A | β-glucosidase | *Thermobifida fusca* |
| **5A** | GH5 | Cel5A | Endoglucanase | *Thermobifida fusca* |
| **6A** | GH6 | Cel6A | Endoglucanase | *Thermobifida fusca* |
| **9A** | GH9 | Cel9A | Processive endo | *Thermobifida fusca* |
| **48A** | GH48 | Cel48A | Exoglucanase | *Thermobifida fusca* |
| **10A** | GH10 | Xyn10A | Xylanase | *Thermobifida fusca* |
| **10B** | GH10 | Xyn10B | Xylanase | *Thermobifida fusca* |
| **11A** | GH11 | Xyn11A | Xylanase | *Thermobifida fusca* |
| **43A** | GH43 | Xyl43A | β-xylosidase | *Thermobifida fusca* |
|  |  |  |  |  |
| **CBM3a** | CBM | CipA | Carbohydrate-Binding Module | *Clostridium thermocellum* |
|  |  |  |  |  |

^a^ The number 2 in subscript (e.g., T_2_ or *t_2_*) specifies that the cohesin or dockerin module is of type II

^b^ Scaffoldin-derived cohesins are numbered according to position from the N-terminus, i.e., Cohesin B1 refers to cohesin number 1 in ScaB, Cohesin C3 refers to cohesin number 3 in ScaC, etc.
