# Supplementary material for: Adaptor Scaffoldins: An Original Strategy for Extended Designer Cellulosomes, Inspired from Nature
Source: mBio. 2016 Apr 5;7(2):e00083-16. doi: 10.1128/mBio.00083-16 (PMC4959524; doi:10.1128/mBio.00083-16)
Supplement: Table S2 — Molecular masses of the recombinant proteins used in this study. [file mbo002162726st2.docx]

**Table S2**: Molecular mass of the recombinant proteins used in this study

**Chimaeric enzymes:**

| **Protein name** | Molecular mass in Da |
| --- | --- |
| ***a-*9A** | 78859 |
| ***b-*48A** | 83433 |
| **5A-*t*** | 42777 |
| **6A-*g*** | 40203 |
|  |  |
| **43A-*c*** | 69878 |
| **11A-*a*** | 43365 |
| **10B-*t*** | 52117 |
| **10a-*f*** | 48626 |

**Chimaeric scaffoldins:**

| **Protein name** | Molecular mass in Da |  | **Protein name** | Molecular mass in Da |
| --- | --- | --- | --- | --- |
| **ScafA** | 36783 |  | **Adaptor1** | 92640 |
| **ScafB** | 38572 |  | **Adaptor2** | 71451 |
| **ScafT** | 36982 |  | **Scaf1** | 74693 |
| **ScafG** | 38427 |  | **Scaf2** | 53893 |
| **ScafC** | 36248 |  | **Scaf3** | 124600 |
| **ScafF** | 35710 |  | **Scaf4** | 126461 |
| **ScafT_2_** | 35710 |  |  |  |
