# Supplementary material for: Adaptor Scaffoldins: An Original Strategy for Extended Designer Cellulosomes, Inspired from Nature
Source: mBio. 2016 Apr 5;7(2):e00083-16. doi: 10.1128/mBio.00083-16 (PMC4959524; doi:10.1128/mBio.00083-16)
Supplement: Table S3 — Primer appendix. [file mbo002162726st3.docx]

**Table S3**: Primers appendix

| Chimeras  (plasmid) | Modules | Primers | Sequence | Restriction enzyme |
| --- | --- | --- | --- | --- |
| 6A-*g*  (pET-28a) | catalytic subunit 6A | Forward | 5’ GGGC**GGTACC**AGAAGAAGCAAAC 3’ | KpnI |
|  |  | Reverse | 5’CAAG**CTCGAG**CTTACCCAGTAAGCCATTC 3’ | XhoI |
|  | dockerin *g* | Forward | 5’GTATT**CCATGG**CACATCACCATCACCATCACGCAGAAGAAGCAAACAAGGG 3’ | NcoI |
|  |  | Reverse | 5’GTCA**GGTACC**GCCTTACCCAGTAAGCCATT 3’ | KpnI |
| Adaptor1  (pET-28a) | CBM3a,  Cohesins  A, B, T^a^ | Forward | 5’AGGT**CCCATGG**CAAATACACCGGTATCAGGCAATTTG 3’ | NcoI |
|  |  | Reverse | 5’GTTCA**AGATCT**TGTTGCATTGCCAACGTTAACAC 3’ | BglII |
|  | dockerin type II  *(C. thermocellum)* | Forward | 5’ TGCACC**GGATCC**AACTAATAAACC TGTAATAGAAG 3’ | BamHI |
|  |  | Reverse | 5’AAAGTC**CTCGAG**CTGTGCGTCGTAATCACTTG 3’ | XhoI |
| Adaptor2  (pET-28a) | Cohesins  A, B, T ^a^ | Forward | 5’CAATTG**CCATGG**GCTCCGATTTACAGGTTGAC 3’ | NcoI |
|  |  | Reverse | 5’ATTGAC**GGATCC**TGTTGCATTGCCAACGTTAAC 3’ | BamHI |
|  | dockerin type II  *(C. thermocellum)* | *see upper case* | | |
| Scaf5^b^  (pET-28a) | Cohesins C,A, CBM3a,  Cohesins T, G^c^ | Forward | 5’GAATAC**CCATGG**GCCTTAAAGTTACAGTAGGAACAG 3’ | NcoI |
|  |  | Reverse | 5’CCAATG**GCTAGC**TTCTTCCTGAGAGACAATCCTG 3’ | NheI |
|  | cohesin F | Forward | 5’AATGGA**GCTAGC**GCCGGTGGTTTATCCGCTGTG 3’ | NheI |
|  |  | Reverse | 5’ATTGCA**GTCGAC**AACAATGATAGCGC  CATCAGTAAG 3’ | SalI |
| Scaf4  (pET-28a) | cohesin T_2_ | Forward | 5’GTTAC**CCATGG**GCGTGGCTCTGGAACTGGATAAG 3’ | NcoI |
|  |  | Reverse | 5’CAACG**GCTAGC**ATAGGAATCTGGAAGCTCTGAAG 3’ | NheI |
|  | Cohesins C, A, CBM3a, Cohesins T,G, F | Forward | 5’CAAGTA**TCTAGA**CTTAAAGTTACAGTAGGAACAG**’**3 | XbaI |
|  |  | Reverse | 5’AATCCA**CTCGAG** AACAATGATAGCGC  CATCAGTAAG 3’ | XhoI |

The different modules were obtained by PCR amplification from relevant genomic DNA except if specified otherwise.

^a^ These fragments were obtained by PCR amplification from the plasmid of *scaf 20L* from our previous report (1)

^b^ Scaf5 was not employed in this study but served for the construction of Scaf4 scaffoldin

^c^ This fragment was obtained by PCR amplification from the plasmid of Scaf3 from our previous report (2)

**References**

1. Vazana Y, et al. (2013) A synthetic biology approach for evaluating the functional contribution of designer cellulosome components to deconstruction of cellulosic substrates. *Biotechnol Biofuels* 6(1):182.

2. Moraïs S, et al. (2012) Deconstruction of lignocellulose into soluble sugars by native and designer cellulosomes. *MBio* 3(6). doi:10.1128/mBio.00508-12.
